# Impact of body mass index on size and composition of urinary stones: a systematic review and meta-analysis

**DOI:** 10.1590/S1677-5538.IBJU.2022.0587

**Published:** 2023-03-30

**Authors:** Daoqi Wang, Jiahong Tan, Erkang Geng, Chuanping Wan, Jinming Xu, Bin Yang, Yuan Zhou, Guiming Zhou, Zhenni Ye, Jiongming Li, Jianhe Liu

**Affiliations:** 1 Department of Urology Kunming Medical University Kunming China Department of Urology, The Second Affiliated Hospital of Kunming Medical University, Kunming, China;; 2 Department of Obstetrics and Gynecology The First People’s Hospital of Yunnan Province Kunming China Department of Obstetrics and Gynecology, The First People’s Hospital of Yunnan Province, Kunming, China

**Keywords:** Urinary Calculi, Body Mass Index, Obesity

## Abstract

**Background:**

Several studies have explored the impact of BMI on size and composition of urinary stones. Because there were controversies, a meta-analysis was necessary to be carried out to provide some evidence of the relationship of BMI and urolithiasis.

**Materials and Methods:**

PubMed, Medline, Embase, Web of Science databases, and the Cochrane Library were searched up to August 12th 2022 for eligible studies. The urolithiasis patients were summarized into two groups: BMI < 25 and ≥ 25 kg/m2. Summary weighted mean difference (WMD), relative risk (RR) and 95% confidence intervals (CI) were calculated through random effects models in RevMan 5.4 software.

**Results:**

A total of fifteen studies involving 13,233 patients were enrolled in this meta-analysis. There was no significant correlation of BMI and size of urinary stone (WMD -0.13mm, 95% CI [-0.98, 0.73], p = 0.77). Overweight and obesity increased the risk of uric acid stones in both genders and in different regions (RR=0.87, [95% CI] = 0.83, 0.91, p<0.00001). There was a higher risk of calcium oxalate stones formation in overweight and obesity group in total patients (RR=0.95, [95% CI] = 0.91, 0.98, p = 0.006). The relationship of BMI and calcium phosphate was not observed in this meta-analysis (RR=1.12, [95% CI] = 0.98, 1.26, p = 0.09). Sensitivity analysis was performed and indicated similar results.

**Conclusions:**

The current evidence suggests a positive association between BMI and uric acid and calcium oxalate stones. It would be of great guiding significance to consider losing weight when treating and preventing urinary stones.

## INTRODUCTION

Urolithiasis is one of the most common diseases encountered in urology with a reported frequency of 7%–13% in North America, 5%–9% in Europe, and 1%–5% in Asia (1, 2). The incidence of urinary stones has increased in both developed and developing countries over the last decades (3). From 1991 to 2000, 2001 to 2010, and 2011 to 2016, the prevalence of urolithiasis in China were 5.95%, 8.86%, and 10.63% (4). The overall prevalence of kidney stones in the USA rose from 3.2% to 10.1% in 1980 to 2016 (5). The five-year recurrence rate of urinary stones has been reported to be between 31.5–50%, and the 20-year recurrence rate is 72% (6, 7). Several factors have been confirmed to be associated with the high prevalence and recurrence of urinary stones, including genetics, age, sex, body mass index (BMI), geographic location, seasonal factors, diet, and occupation (8, 9). Although many methods could be performed to remove urinary stones, urolithiasis was not cured. The etiological treatment of most urolithiasis can’t be conducted due to the lack of detailed mechanism of urinary stones formation (9, 10).

Many studies indicated that urolithiasis is a systemic disorder and related to metabolic syndrome (11-13). The higher prevalence of urinary stones is found in people with higher BMI (14-16). Overweight and obesity have been investigated to increase the risk of urolithiasis (17-19). There was a study indicating the increased rate and decreased time of stones recurrence in those obese first-time stone formers (20).

Body size has been found to be associated with not only the incidence of urolithiasis but also the size and composition of urinary stones, although the mechanisms involved have not been clarified. Several studies have been conducted to explore the effect of BMI on size and composition of urinary stones in the past two decades (14, 19-32). Moreover, in view of the inconsistent findings of the studies reported to date, a meta-analysis was necessary to assess the evidence for a relationship between BMI and urolithiasis.

## MATERIALS AND METHODS

### Search strategy

The systematic literature search was conducted on PubMed, Medline, Embase, Web of Science databases, and the Cochrane Library, following the standard criteria for reporting meta-analysis, up to August 12th 2022 for eligible studies published from 2000 (33). The search terms were: [(urolithiasis or lithiasis or nephrolithiasis or calculus or calculi or stone or stones) AND (overweight or obese or obesity or body mass index or BMI)]. Two reviewers screened all the titles and abstracts independently. The language was restricted to English, and articles studying the impact of body size on size and composition of urinary stones were included for further screening. We conducted this meta-analysis according to PRISMA 2020 (Preferred Reporting Items for Systematic Reviews and Meta-Analyses 2020) (34).

### Inclusion criteria and exclusion criteria

Inclusion criteria: (1) The body size should be classified by BMI, and the BMI classification could be summarized into two groups which were BMI < 25 and ≥ 25 kg/m^2^. (2) The size and composition of urinary stones should be compared by BMI. (3) The full text was accessible online. (4) Studies should report at least one of relevant clinical outcomes of interest (described in data extraction part).

Exclusion criteria: (1) Studies were not in English. (2) Conference abstracts. (3) The interesting data could not be extracted or calculated.

Two reviewers conducted this studies selection process independently. A discussion was conducted when disagreement arose. If disagreement persisted, a third investigator was consulted to reach a consensus.

### Study quality and level of evidence

The level of evidence of each study was evaluated via the criteria provided by the Oxford Center for Evidence-Based Medicine (35). The methodological quality of the non-randomized studies included in this meta-analysis was assessed by Newcastle Ottawa Scale (36). The detailed assessment was summarized in Supplementary Table-1.

Two reviewers carried out this assessment procedure independently and reached a consensus through discussion if disagreements appeared.

### Data extraction

The following data were extracted by two reviewers independently using a predetermined data extraction form, including the first author, year of publication, nation, number of samples, classification for BMI, age and sex ratio of patients. The basic characteristics of patients included the level of serum calcium and urate, urine pH, the volume of 24h-urine, calcium, oxalate, urate and citrate excretion in 24h-urine. The interesting outcomes included size of urinary stones, the composition of urinary stones, such as calcium oxalate, calcium phosphate, uric acid, carbapatite and cystin. The data of mixed urinary stones were also extracted. The mixed urinary stones represented more than one composition of stones described in original research.

### Statistical Analysis

The meta-analysis was conducted using Review Manager software (RevMan version 5.4; Cochrane Collaboration, London, UK). All unit of the urine volume was unified to mL and other measurements to mg/day to reduce the heterogeneity and make it easier to be calculated and analyzed. The classifications of BMI were summarized into two groups: BMI < 25 and BMI ≥ 25 kg/m^2^ according to the guidelines of the Cochrane Collaboration (37). The data were extracted and analyzed, including in subgroups based on sex (male or female) and geographic region (Asia, North America, or Europe). Weighted mean difference (WMD) was used for the continuous data and relative risk (RR) for the dichotomous data. All the results are represented with 95% confidence intervals (95% CI). The heterogeneity among studies was assessed by the Chi-square test and I^2^ value. The p > 0.05 or I^2^ < 50% were considered as good homogeneity. The pooled effects were analyzed by the z test, and p < 0.05 represented statistical significance. Publication bias was assessed using funnel plots. The sensitivity analysis was performed using selected studies with a high score (scored ≥7) according to Newcastle–Ottawa Scale.

## RESULTS

### Characteristics and methodological quality of included studies

**Figure 1:**
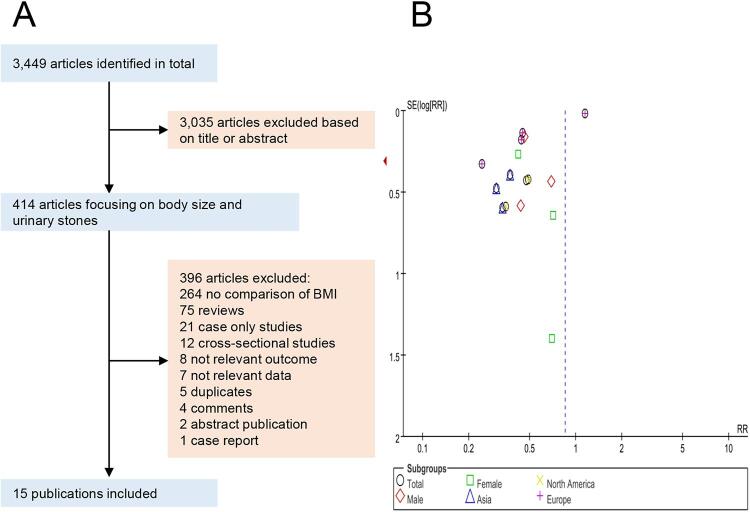
– A) Flow-chart of study selection. B) Funnel plot of uric acid.

The literature search and study selection processes are shown in Figure-1A. A total of 15 studies (13,233 patients) were included in the analysis. These studies were conducted across the World, seven studies in Asia, four studies in Europe, three studies in North America, and one study in South America. There were three cohort studies rated as level 2b of evidence and twelve case-control studies rated as level 3b (shown in Table-1). The full stars given to the methodological quality of a study were nine stars according to the Newcastle Ottawa Scale. In the three cohort studies, all studies did not select the non-exposed cohort in the same community and did not control for any additional factor, and one study did not conduct adequate follow-up of cohorts. Therefore, two cohort studies got seven stars and one got six stars (shown in Supplementary Table-1). In the twelve case-control studies, all studies did not select controls in the same community and did not describe non-response rate, and one study did not select representative cases. Therefore, eleven case-control studies got seven stars and one got six stars (shown in Supplementary Table-1). Studies scored ≥ 7 stars were considered to be of high methodological quality to be selected for sensitivity analysis.

**Table 1:** Characteristics and methodological quality of included studies.

Studies	Nation	LOE	Study quality	Number of samples	BMI (kg/m2) Classification (Number of each group)	Age (years) (M± SD)	Sex (male: female)
Takeuchi H,. 2019 [14]	Japan	2b	7/9	63	BMI<25: (36) BMI≥25: (27)	55.6±16	49:14
Trinchieri A, et al. 2016 [19]	Italy	3b	7/9	1698	BMI<18.5: (91) 18.5≤BMI<24.9: (924) 25≤BMI<29.9: (542) 30≤BMI: (141)	45.9±14.6	984:714
Lee SC, et al. 2007 [20]	South Korea	3b	7/9	704	BMI<25: (475) BMI≥25: (229)	42.8±13.2	470:234
Ekeruo WO, et al. 2004 [21]	The USA	3b	7/9	1021	BMI<25: (881) BMI≥25: (140)	53.2±14.9	ND
Daudon M, et al. 2006(1) [22]	France	3b	7/9	1930	BMI<25: (1259) 25≤BMI<30: (480) 30≤BMI: (191)	ND	1370:561
Daudon M, et al. 2006(2) [23]	France	3b	7/9	2464	BMI<25: (1416) 25≤BMI<30: (703) 30≤BMI: (345)	53.6±11.4	1760:704
Chou YH, et al. 2010 [24]	Taiwan, China	3b	7/9	907	18.5≤BMI<25: (251) 25≤BMI<27: (304) 27≤BMI: (352)	53.9±14	661:246
del Valle EE, et al. 2010 [25]	Argentina	3b	7/9	817	BMI<24.9: (337) 25≤BMI<29.9: (322) 30≤BMI: (158)	ND	459:358
Mosli HA, et al. 2012 [26]	Saudi Arabia	2b	7/9	173	BMI<18: (5) 18.5≤BMI<24.9: (30) 25≤BMI<30: (64) 30≤BMI: (24)	46.03±12.7	131:42
Al-Hayek S, et al. 2013 [27]	The USA	3b	7/9	325	BMI<25: (88) 25≤BMI<30: (103) 30≤BMI: (134)	51.8±12.5	162:163
Najeeb Q, et al. 2013 [28]	India	3b	7/9	100	BMI<25: (28) 25≤BMI<30: (38) 30≤BMI: (34)	38.49±13.72	70:30
Çaltık Yılmaz A, et al. 2015 [29]	Turkey	2b	6/9	84	BMI<18: (52) 18≤BMI<25: (20) 25≤BMI: (12)	ND	42:42
Fram EB, et al. 2015 [30]	The USA	3b	7/9	382	BMI<25: (79) 25≤BMI<30: (140) 30≤BMI: (163)	46.4±15	224:382
Shavit L, et al. 2014 [31]	The UK	3b	6/9	2132	BMI<25: (833) 25≤BMI<30: (863) 30≤BMI: (436)	46±15	1503:629
Almannie RM, et al. 2019 [32]	Saudi Arabia	3b	7/9	433	BMI<18: (24) 18≤BMI<25: (81) 25≤BMI: (328)	ND	316:117

LOE: level of evidence; BMI: body mass index; ND: not demonstrated.

The classifications of BMI were more than two groups in several studies (shown in Table-1). The ratio of BMI < 25 to BMI ≥ 25 kg/m^2^ was 0.941 after summarizing the classifications of BMI into two groups which were BMI < 25 and BMI ≥ 25 kg/m^2^. The average age of patients in eleven studies were 49.282 years old. And the ratio of male to female was 1.936. All detailed characteristics of selected studies are shown in Table-1.

### Publication bias

The publication bias was detected using funnel plots. As showed in Figure-1B, the funnel plot of uric acid stones including the most studies seemed asymmetric, suggesting that there was a publication bias in this meta-analysis.

### Characteristics of serum and 24h-urine chemistries

The meta-analysis also included several serum and 24-h urinary biochemical parameters. The results indicated that the level of serum calcium and urate was higher in BMI ≥ 25 kg/m^2^group compared to BMI < 25 kg/m^2^ group. The volume of 24h-urine in BMI ≥ 25 kg/m^2^ group was more than that in BMI < 25 kg/m^2^ group. The pH value of 24h-urine was lower in BMI ≥ 25 kg/m^2^ group compared to BMI < 25 kg/m^2^ group. And all the calcium, oxalate, urate and citrate excretion in 24h-urine in BMI ≥ 25 kg/m^2^group were more than those in BMI < 25 kg/m^2^ group. All the differences were statistically significant (Table-2). The detailed characteristics of serum and 24h-urine chemistries are summarized in Table-2.

**Table 2:** Characteristics of serum and 24h-urine chemistries of the patients.

	Characteristics	Studies	Number of patients	Heterogeneity		Overall effect	
			BMI<25 vs BMI≥25(kg/m^2^)	p value	I2 (%)	WMD (95% CI)	p value*
Serum	Calcium (mg/dL)	[20, 28]	503/301	0.95	0	-0.10 (-0.19, -0.01)	**0.03**
	Urate (mg/dL)	[20, 28, 30]	582/604	0.02	75%	-0.86 (-1.04, -0.68)	**<0.00001**
24-urine	Volume (mL)	[19, 20, 30]	1362/1069	0.4	0	-88.49 (-148.02, -28.95)	**0.004**
	pH	[14, 19, 20, 28, 31]	2180/2164	<0.00001	98%	0.13(0.09, 0.16)	**<0.00001**
	Calcium excretion (mg/day)	[20, 30, 31]	1387/1831	0.26	26%	-11.47(-19.97, -2.96)	**0.008**
	Oxalate excretion (mg/day)	[20, 30, 31]	1387/1831	0.58	0	-1.62(-2.67, -0.57)	**0.003**
	Urate excretion (mg/day)	[14, 20, 30, 31]	1423/1858	0.006	76%	-88.23(-101.87, -74.59)	**<0.00001**
	Citrate excretion (mg/day)	[20, 30, 31]	1387/1831	0.1	57%	-33.28(-52.16, -14.40)	**0.00006**

WMD: weighted mean difference, CI: confidence interval

*p <0.05 was considered statistically significant and shown in bold.

### Size of urinary stones

**Figure 2:**
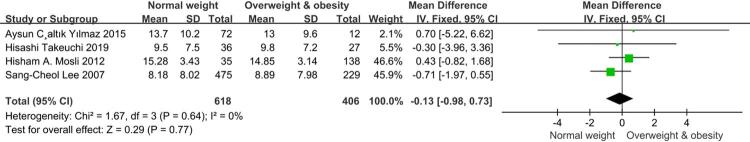
Forest plots of size of urinary stones.

There were four studies selected for meta-analysis of the size of urinary stones. The results indicated no significant difference in size of urinary stones between the BMI < 25 and ≥ 25 kg/m^2^ group (WMD -0.13mm, 95% CI [-0.98, 0.73], p = 0.77). Forest plots are shown in Figure-2.

### Calcium oxalate

**Figure 3:**
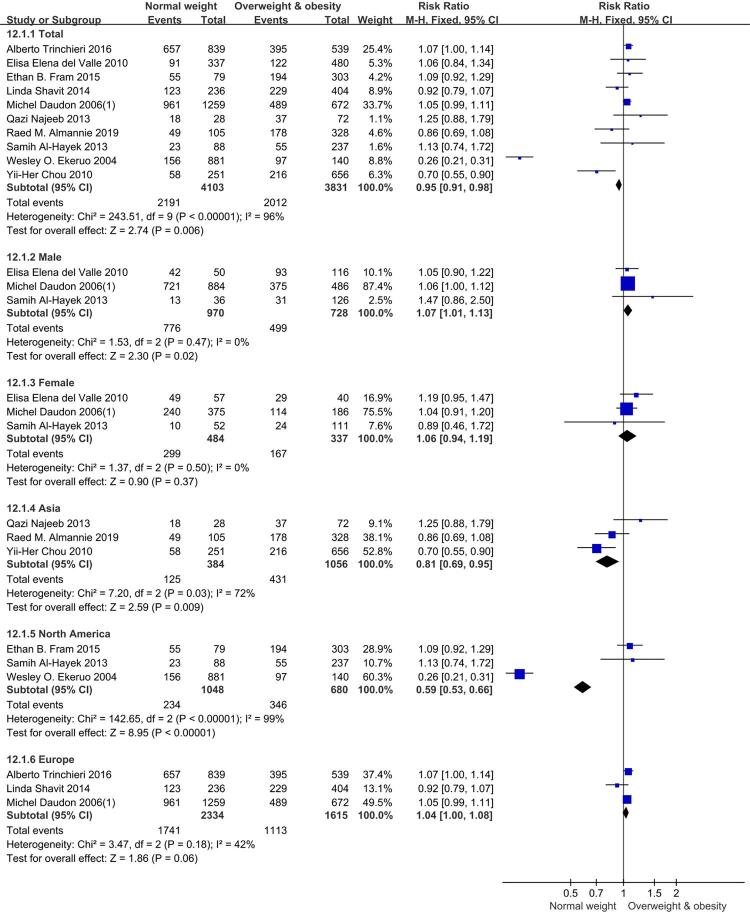
Forest plots of calcium oxalate.

A total of ten studies were enrolled in this meta-analysis regarding calcium oxalate. As shown in Figure-3, those in BMI ≥ 25 kg/m^2^ group had a higher risk, RR=0.95, [95% CI] = 0.91, 0.98, p = 0.006. However, when gender was considered, the trend was opposite. Both in male and female subgroups, the results indicated a lower risk in BMI ≥ 25 kg/m^2^ group compared to BMI<25 kg/m^2^ group. In male subgroup, RR=1.07, [95% CI] = 1.01, 1.13, p = 0.02. In female subgroup, the differences were not statistically significant, RR=1.06, [95% CI] = 0.94, 1.19, p =0.37. There were vary trends in different regions. In both Asia and North America subgroups, those in BMI ≥ 25 kg/m^2^ group had a higher risk, in Asia subgroup, RR= 0.81, [95% CI] = 0.69, 0.95, p =0.009, in North America, RR= 0.59 [95% CI] =0.53, 0.66, p<0.00001. But in Europe subgroup, there was no significant difference between BMI ≥ 25 kg/m^2^ group and BMI<25 kg/m^2^ group, RR=1.04, [95% CI] = 1.00, 1.08, p=0.06. Forest plots of groups and subgroups are shown in Figure-3.

### Calcium phosphate

**Figure 4:**
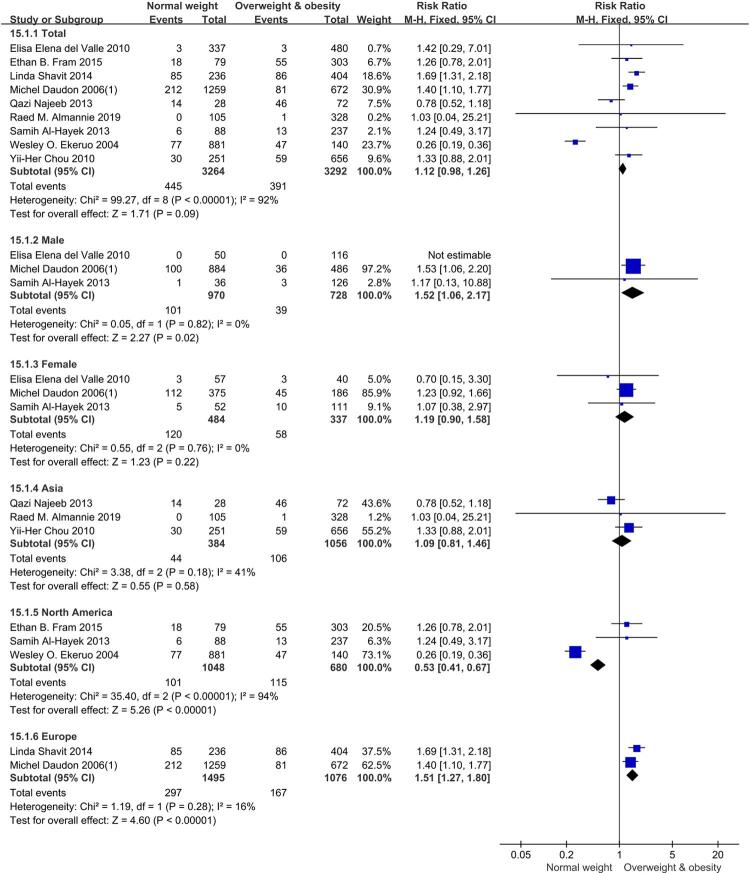
Forest plots of calcium phosphate.

There was no significant difference of calcium phosphate formation between BMI ≥ 25 kg/m^2^ group and BMI<25 kg/m^2^ group in total patients according to the meta-analysis involving nine eligible studies (RR=1.12, [95% CI] = 0.98, 1.26, p = 0.09). In male subgroup, those in BMI≥25 kg/m^2^ group had a lower risk, RR=1.52, [95% CI] =1.06, 2.17, p = 0.02. Female subgroup showed no significant difference, RR=1.19, [95% CI] = 0.90, 1.58, p = 0.22. However, the differences were statistically significant when region factor was considered. The trends were opposite in North America and Europe subgroups. In North America subgroup, there was a higher risk in BMI≥25 kg/m^2^ group, RR=0.53, [95% CI] = 0.41, 0.67, p <0.00001. In Europe subgroup, those in BMI≥25 kg/m^2^ group had a lower risk, RR=1.51, [95% CI] =1.27, 1.80, p <0.00001. But in Asia subgroup, the difference was not statistically significant, RR=1.09, [95% CI] =0.81, 1.46, p =0.58. Forest plots of groups and subgroups are shown in Figure-4.

### Uric acid

**Figure 5:**
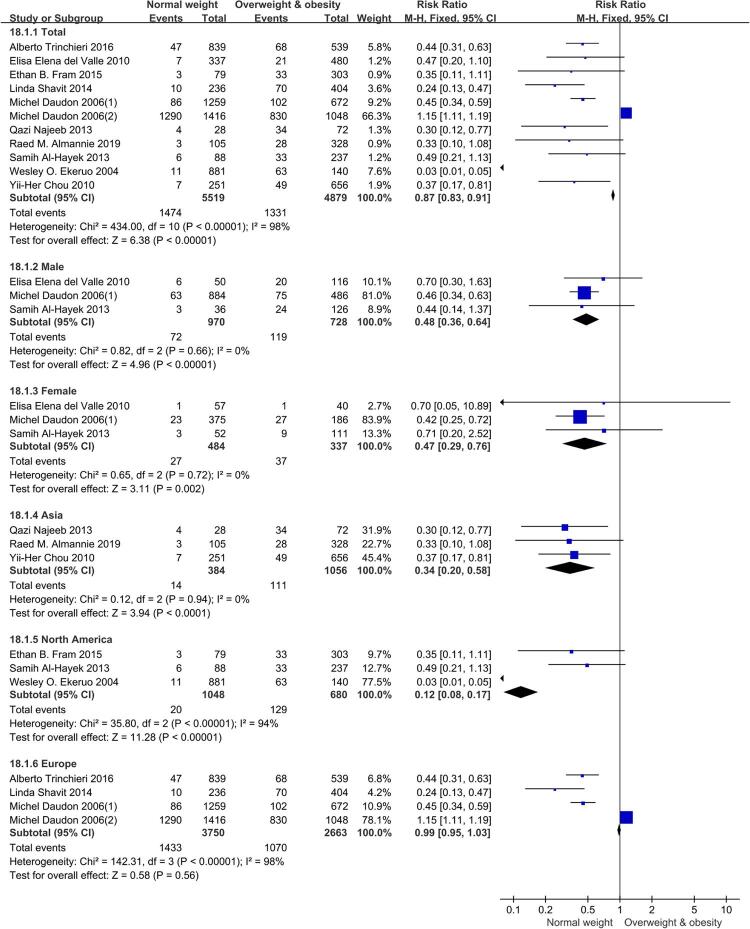
Forest plots of uric acid.

Those in BMI≥25 kg/m^2^ group had a higher risk of uric acid in nearly all groups and subgroups except Europe subgroup based on this meta-analysis involving eleven relevant studies. In total patients, RR=0.87, [95% CI] = 0.83, 0.91, p<0.00001. In male subgroup, RR=0.48, [95% CI] =0.36, 0.64, p<0.00001. In female subgroup, RR=0.47, [95% CI] =0.29, 0.76, p=0.002. In Asia subgroup, RR=0.34, [95% CI] =0.20, 0.58, p<0.00001. In North America subgroup, RR=0.12, [95% CI] =0.08, 0.17, p<0.00001. However, in Europe subgroup, the difference was not statistically significant, RR=0.99, [95% CI] =0.95, 1.03, p=0.56. Forest plots of groups and subgroups are shown in Figure-5.

### Carbapatite

**Figure 6:**
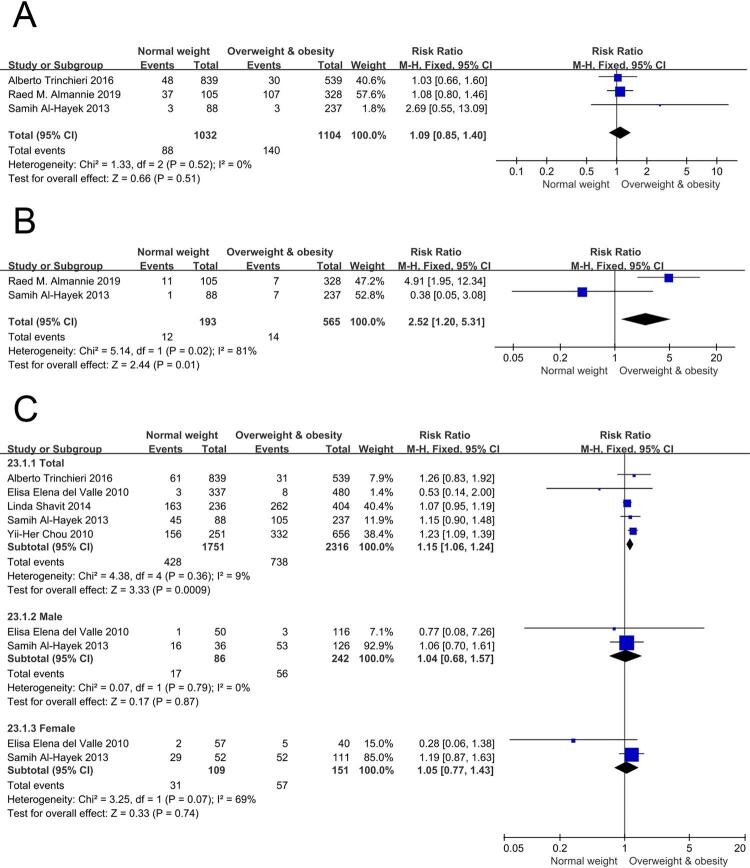
– A) forest plots of carbapatite. B) forest plots of cystin. C) forest plots of mixed stones.

Meta-analysis of carbapatite showed that there was no significant difference between the BMI < 25 and ≥ 25 kg/m^2^ group. (RR= 1.09, [95% CI] = 0.85, 1.40, p =0.66). Forest plots are shown in Figure-6A.

### Cystin

The results of meta-analysis of cystin indicated a lower risk in BMI≥25 kg/m^2^ group compared to BMI<25 kg/m^2^ group (RR= 2.52 [95% CI] =1.20, 5.31, p=0.01). Forest plots are shown in Figure-6B.

### Mixed stones

A total of five eligible studies were involved in this meta-analysis of mixed stones. The results indicated a lower risk in BMI≥25 kg/m^2^ group compared to BMI<25 kg/m2 group, RR=1.15, [95% CI] = 1.06, 1.24, p =0.0009. However, in both male and female subgroups, there were no significant differences. In male subgroup, RR=1.04, [95% CI] = 0.68, 1.57, p =0.87. In female subgroup, RR=1.05, [95% CI] = 0.77, 1.43, p =0.74. Forest plots of groups and subgroups are shown in Figure-6B.

### Sensitivity analysis

The detailed characteristics of size and composition of urinary stones in the patients were summarized in Table-3. All studies scored ≥ seven stars according to Newcastle–Ottawa Scale were enrolled in this sensitivity analysis (summarized in Table-4), and the outcomes of size of urinary stones, calcium oxalate, calcium phosphate, uric acid, carbapatite, cystin and mixed stones were stable, demonstrating that this meta-analysis was reliable.

**Table 3:** Characteristics of size and composition of urinary stones in the patients.

Items	Studies	Number of patients	Heterogeneity		Overall effect	
		BMI<25 vs BMI≥25 (kg/m2)	p value	I2 (%)	RR/WMD (95% CI)	p value*
size of urolithiasis	[14, 20, 26, 29]	618/406	0.64	0	-0.13(-0.98, 0.73)	0.77
calcium oxalate	[19, 21, 22, 24, 25, 27, 28, 30-32]	4103/3831	<0.00001	96%	0.95(0.91, 0.98)	**0.006**
calcium oxalate (male)	[22, 25, 27]	970/728	0.8	0%	1.34(1.05, 1.72)	**0.02**
calcium oxalate (female)	[22, 25, 27]	484/337	0.32	13%	1.16(0.85, 1.58)	0.36
calcium phosphate	[21, 22, 24, 25, 27, 28, 30-32]	3264/3292	<0.00001	91%	1.15(0.97, 1.35)	0.1
calcium phosphate(male)	[22, 25, 27]	970/728	0.82	0%	1.52(1.06, 2.17)	**0.02**
calcium phosphate(female)	[22, 25, 27]	484/337	0.76	0%	1.19(0.90, 1.58)	0.22
uric acid	[19, 21, 22, 23, 24, 25, 27, 28, 30-32]	5519/4879	0.00001	98%	0.87(0.83, 0.91)	**<0.00001**
uric acid (male)	[22, 25, 27]	970/728	0.66	0%	0.48(0.36, 0.64)	**<0.00001**
uric acid (female)	[22, 25, 27]	484/337	0.72	0%	0.47(0.29, 0.76)	**0.002**
carbapatite	[19, 27, 32]	1032/1104	0.52	0%	1.09(0.85, 1.40)	0.51
cystin	[27, 32]	193/565	0.02	81%	2.52(1.20, 5.31)	**0.01**
mixed stones	[19, 24, 25, 27, 31]	1751/2316	0.36	9%	1.15(1.06, 1.24)	**0.00009**
mixed stones (male)	[25, 27]	86/242	0.79	0%	1.04(0.68, 1.57)	0.87
mixed stones (female)	[25, 27]	109/151	0.07	69%	1.05(0.77, 1.43)	0.74

*p <0.05 was considered statistically significant and shown in bold.

**Table 4:** Sensitivity analysis.

Items	Studies	Number of patients	Heterogeneity		Overall effect		Higher in
		BMI<25 vs BMI≥25 (kg/m^2^)	p value	I2 (%)	RR/WMD (95% CI)	p value*	
size of urinary stones	14, 20, 26]	546/394	0.45	0	-0.15(-1.01, 0.72)	0.74	/
calcium oxalate	[19, 21, 22, 24, 25, 27, 28, 30, 32]	3867/3427	<0.00001	97%	0.95(0.91, 0.99)	**0.01**	BMI ≥25
calcium phosphate	[21, 22, 24, 25, 27, 28, 30, 32]	3028/2888	<0.00001	91%	0.98(0.85, 1.14)	0.83	/
uric acid	[19, 21, 22, 23, 24, 25, 27, 28, 30, 32]	5283/4475	0.00001	98%	0.89(0.86, 0.93)	**<0.00001**	BMI ≥25
carbapatite	[19, 27, 32]	1032/1104	0.52	0%	1.09(0.85, 1.40)	0.51	/
cystin	[27, 32]	193/565	0.02	81%	2.52(1.20, 5.31)	**0.01**	BMI <25
mixed stones	[19, 24, 25, 27]	1515/1912	0.63	0%	1.20(1.08, 1.34)	**0.001**	BMI <25

*p <0.05 was considered statistically significant and shown in bold.

## DISCUSSION

The incidence of urolithiasis is increasing worldwide, leading to physical and financial burden (1-3). At present, the treatment of urolithiasis is usually limited to remove stones, due to the lack of knowledge of etiology and mechanism of stones formation in most urolithiasis patients. Investigation of the common and modifiable risks of urolithiasis may get insight in the pathogenesis of urinary stones and explore new approaches to treatment and prevention. Overweight and obesity are also becoming a global problem and are known to have a role in the development of several chronic diseases, such as hypertension, diabetes, cancers, chronic kidney disease, and urolithiasis (38). The incidence of urinary stones is significantly increased in patients with high BMI (17-20). The effect of body size on urinary stones formation is not clear yet. This meta-analysis is the first systematic review focusing on the impact of BMI on the size and composition of urinary stones, exploring how overweight and obesity contribute to urinary stones formation.

In this meta-analysis, the average age was 49.282 years which was older than peak age of 20-40 years reported by previous studies (39). The morbidity of urolithiasis in males was near two times more than that in females in this meta-analysis, indicating the high incidence of urolithiasis in males. But recent studies have demonstrated the increased prevalence of urolithiasis in females, and the male-to-female ratio has decreased from 3:1 to 1.3:1 between 1970 and 2000 (40). Moreover, medical care utilization due to urolithiasis increased 52% among women whereas only 22% among men (41). The reason underlying this change is not clear now. There are several hypothesizes. The change of society role and workplace in females might result in dietary and lifestyle changes which could contribute to urinary stones formation. For example, one study found that women tended to drink less water than men (42). Another hypothesis was that the increased prevalence of obesity in females was higher than that in males, and high BMI has been demonstrated as a risk factor for urolithiasis. Moreover, overweight and obesity in females had a larger impact on the development of urolithiasis, with OR=1.35, [95% CI] =1.33, 1.37 in females, and OR=1.04, [95% CI] =1.02,1.06 in males (43).

In this meta-analysis, we found a higher level of serum calcium, calcium excretion and oxalate excretion in 24h-urine in overweight and obesity group. However, there were conflict results of serum calcium in other studies. Zahra Jafari-Jafari-Giv, et al. reported a lower level of serum calcium in obese people, while Wang, et al. found that there was no association between serum calcium and body size (44, 45). Further investigations are warranted to explore the relationship of serum calcium and BMI. In this meta-analysis, we also found a higher risk of calcium oxalate urinary stones in overweight and obesity group. The calcium oxalate accounted for approximately 80% urinary stones (46). Supersaturation of calcium oxalate in urine was a major contribution to formation of calcium oxalate stones (47). High level of urine urate was also the risk factor of calcium oxalate stones formation, because high concentration of urate could decrease the solubility of calcium oxalate and reduce inhibitory activity of glycosaminoglycans on the crystallization of calcium oxalate, promoting the formation of calcium oxalate stones (48). Obese individuals were more likely to have hyperuricosuria, hyperoxaluria and hypercalciuria, because those people usually had a high intake of calories, calcium, animal protein, and sodium. Therefore, overweight, and obese people had a high risk of calcium oxalate stones. And several studies indicated that diets with high fruits and vegetables and low protein and salt were associated with decreased calcium oxalate supersaturation (49-51). However, considering gender, the trend was opposite in both male and female subgroups. The limited samples and publication bias might be the reasons of this opposite trend. In the funnel plot (Supplementary Figure-1), we could found the plots were located at the bottom of the funnel and nearly almost plots were on the right side of the axis representing ration 1. In Asia and North America subgroups, overweight and obese individuals had high risk of calcium oxalate, but in Europe subgroup, there was no significant difference. The trend of calcium oxalate stones in overweight and obese people among these regions varies considerably on account of environmental factors, especially dietary intake, and lifestyle (52). In general, high BMI was a risk factor of calcium oxalate stones formation, but different dietary intake and lifestyle might have impact on this type of urinary stones.

Our results indicated that there was no significant difference of calcium phosphate between BMI < 25 and BMI ≥ 25 kg/m^2^ groups in this meta-analysis. But the general trend was that higher BMI tended to lower percentage of calcium phosphate stones, except in North America subgroup. It was interesting that obesity appears to affect potential lithogenic factors including oxalate and uric acid, but not calcium (53, 54). The development of calcium phosphate stones was associated more with calcium metabolism factors such as hyperparathyroidism, which might be the reason why the prevalence of calcium phosphate stones is not higher in obese subjects (24). Further study is needed to explore the exact mechanism underlying the relationship of calcium phosphate stones and BMI.

In this meta-analysis, the results demonstrated that there was a strong relationship between formation of uric acid stones and BMI; overweight and obese individuals tended to be more likely to develop uric acid stones independent of sex or region. The level of serum urate and urate excretion in 24h-urine were also increased in overweight and obesity groups. Those obese people might have increased dietary purine intake, contributing to the high level of serum urate and urine urate and were more likely to have hyperinsulinemia or insulin resistance damaging the renal function in ammonium production and the ability to excrete acid, and thus decreasing urine pH (55, 56). The results also indicated a lower pH in 24h-urine in high BMI group in our analysis. The acidic environment in urine could contribute to the formation of uric acid stones. Hyperinsulinemia could also lead to increased urinary excretion of uric acid which was an important risk factor for uric acid stones formation (57).

We also analyzed the formation of carbapatite, cystin and mixed stones in normal weight and overweight or obesity groups. Only three studies were eligible for analysis of carbapatite and two for cystin. There was no significant difference in the frequency of carbapatite stones according to BMI. It has been reported that carbapatite stones are more closely associated with sex than with BMI. Carbapatite and struvite stones have been found to be more common in women (57), whereas cystin stones are associated with genetic factors. Cystinuria is caused by a failure in proximal tubular reabsorption of filtered cystine, which is a homodimer of the amino acid cysteine. Cystinuria is an autosomal recessive genetic disorder caused by two genes (i.e., SLC3A1 and SLC7A9). Most patients with cystinuria presented in childhood with recurrent urinary stones and cystinuria (58). Our meta-analysis of the only two eligible studies found a lower incidence of cystin stones in the group with a high BMI. One of the two studies, reported by Almannie et al., had a much larger sample size than the other and showed a high incidence of cystin stones in a normal weight group, which the authors could not explain (32). In reality, cystin stones are more likely to form in urine in an acidic environment. Therefore, alkaline urine with a pH in the range of 7.0–7.5 would reduce the solubility of cystine and prevent recurrence of cystin stones (59). Our meta-analysis found that individuals who were overweight or obese were at lower risk of mixed stones, which meant that they tended to have a single urinary stone. However, overweight and obese individuals were more likely to have high urate excretion and low pH in urine, which were risk factors for uric acid stones. The high level of urate in urine also increased the saturation of calcium oxalate in urine. Therefore, high BMI should have more mixed urinary stones at least mixture of uric acid and calcium phosphate, which was opposite to the results in this meta-analysis. Further studies are necessary to explore the relationship of mixed stone and BMI.

There were some limitations in this meta-analysis. First, not all the selected studies had the information of characteristics in serum and 24h-urine which were important for explaining formation of urolithiasis. Second, there were significant heterogeneities when assessing some data in total samples. Third, the eligible studies for subgroups analysis were limited, which might have publication bias and influence the results.

## CONCLUSION

This meta-analysis demonstrated that overweight and obesity increase the risk of uric acid stones in both sexes and in different regions and that the risk of calcium oxalate formation is increased in overweight and obese patients. Weight loss should be considered in the prevention and treatment of uric acid and calcium oxalate stones.

## APPENDIX

Supplementary Table 1 - Methodological quality of the included non-randomized studies using Newcastle-Ottawa Quality Assessment Scale.


