# Supplementary material for: Impact of body mass index on size and composition of urinary stones: a systematic review and meta-analysis
Source: Int Braz J Urol. 2023 Mar 30;49(3):281–98. doi: 10.1590/S1677-5538.IBJU.2022.0587 (PMC10335896; doi:10.1590/S1677-5538.IBJU.2022.0587)
Supplement: Supplementary file 1 [file 1677-6119-ibju-49-03-0281-suppl01.pdf]

APPENDIX

Supplementary Table 1 - Methodological quality of the included non-randomized studies using Newcastle-Ottawa Quality Assessment Scale.

	Selection				Comparability	Expouse			
Case-Control Studies	Is the case definition adequate?	Representative-ness of the cases	Selection of Controls	Definition of Controls	Comparability of cases and controls on the basis of the design or analysis	Ascertain-ment of exposure	Same method of ascertainment for cases and controls	Non-Response Rate	Total Score
Trinchieri A, et al. 2017 [19]	✭	✭	/	✭	✭	✭	✭	/	7
Lee SC, et al. 2008 [20]	✭	✭	/	✭	✭	✭	✭	/	7
Ekeruo WO, et al. 2004 [21]	✭	✭	/	✭	✭	✭	✭	/	7
I Daudon M, et al. 2006(1) [22]	✭	✭	/	✭	✭	✭	✭	/	7
Daudon M, et al. 2006(2) [23]	✭	✭	/	✭	✭	✭	✭	/	7
Chou YH, et al. 2010 [24]	✭	✭	/	✭	✭	✭	✭	/	7
del Valle EE, et al. 2010 [25]	✭	✭	/	✭	✭	✭	✭	/	7
Al-Hayek, S, et al. 2013 [27]	✭	✭	/	✭	✭	✭	✭	/	7
Najeeb Q, et al. 2013 [28]	✭	✭	/	✭	✭	✭	✭	/	7
Fram EB, et al. 2015 [30]	✭	✭	/	✭	✭	✭	✭	/	7
Shavit L, et al. 2014 [31]	✭	/	/	✭	✭	✭	✭	/	6
Almannie RM, et al. 2019 [32]	✭	✭	/	✭	✭	✭	✭	/	7

	Selection				Comparability	Outcome			
Cohort Studies	Representa-tiveness of the exposed cohort	Selection of the non-exposed cohort	Ascertain-ment of exposure to implants	Demons-tration that outcome of interest was not present at start of study	Comparability of cohorts on the basis of the design or analysis	Assessment of outcome	Was follow up long enough for outcomes to occur	Adequacy of follow up of cohorts	Total Score
Takeuchi H, et al. 2019 [14]	✭	/	✭	✭	✭	✭	✭	✭	7
Mosli, HA, et al. 2012 [26]	✭	/	✭	✭	✭	✭	✭	✭	7
Caltık Yılmaz, A, et al. 2015 [29]	✭	/	✭	✭	✭	✭	✭	/	6
